# A perspective on FAIR quality control in multiplexed imaging data processing

**DOI:** 10.3389/fbinf.2024.1336257

**Published:** 2024-02-09

**Authors:** Wouter-Michiel A. M. Vierdag, Sinem K. Saka

**Affiliations:** Genome Biology Unit, European Molecular Biology Laboratory (EMBL), Heidelberg, Germany

**Keywords:** multiplexed imaging, image data, image analysis, quality control, FAIR data

## Abstract

Multiplexed imaging approaches are getting increasingly adopted for imaging of large tissue areas, yielding big imaging datasets both in terms of the number of samples and the size of image data per sample. The processing and analysis of these datasets is complex owing to frequent technical artifacts and heterogeneous profiles from a high number of stained targets To streamline the analysis of multiplexed images, automated pipelines making use of state-of-the-art algorithms have been developed. In these pipelines, the output quality of one processing step is typically dependent on the output of the previous step and errors from each step, even when they appear minor, can propagate and confound the results. Thus, rigorous quality control (QC) at each of these different steps of the image processing pipeline is of paramount importance both for the proper analysis and interpretation of the analysis results and for ensuring the reusability of the data. Ideally, QC should become an integral and easily retrievable part of the imaging datasets and the analysis process. Yet, limitations of the currently available frameworks make integration of interactive QC difficult for large multiplexed imaging data. Given the increasing size and complexity of multiplexed imaging datasets, we present the different challenges for integrating QC in image analysis pipelines as well as suggest possible solutions that build on top of recent advances in bioimage analysis.

## 1 Introduction

With the advancements in multiplexed imaging and spatial omics technologies, researchers now have access to large imaging datasets made up of many channels (in direct or combinatorial fashion) for visualization of many different proteins, RNA, or DNA targets *in situ* (Hickey et al., 2022; Moffitt et al., 2022). In their current state, these technologies enable distinguishing cell types and provide insights into tissue architecture and cellular function in healthy tissues and under disease conditions. For this, several large consortia such as the Human BioMolecular Atlas Program (HuBMAP) and Human Tumor Atlas Network (HTAN) have been commissioned to create atlases by integrating data from highly multiplexed imaging technologies with single-cell genomics and transcriptomics data (HuBMAP consortium, 2019; Rajewsky et al., 2020; Rozenblatt-Rosen et al., 2020). These techniques are also starting to be applied to cohorts of clinical cancer samples to reveal new insights about disease prognosis and treatment response (Schürch et al., 2020; Wang et al., 2021; Lin et al., 2023).

While providing unprecedented potential to study the state of cells within their spatial context, the scale and dimensionality of the imaging data make analysis complex and time-consuming (Alexandrov et al., 2023). To this end, multiple semi-automated end-to-end workflows have been established such as MCMICRO, Steinbock, and Mplexable (Schapiro et al., 2021; Windhager et al., 2021; Eng et al., 2022). These workflows encompass essential processing steps such as illumination correction, stitching and registration, intensity projection, segmentation, feature extraction, and cell type annotation. Each of these steps can be prone to introduce errors that might affect the downstream analysis. For example, despite large improvements in supervised deep learning instance segmentation methods for nucleus and cell segmentation in diverse settings and without fine-tuning of models (such as Greenwald et al., 2022; Pachitariu and Stringer, 2022), segmentation instances can still incorrectly merge or separate objects to be segmented, particularly in dense tissue areas. Hickey *et al.* showed that segmentation noise in areas like this can negatively affect cell type annotation (Hickey et al., 2021). This is particularly the case for tissues on which the model was not trained or when cells are shaped differently from what the model has learned. While incorrect cell type annotations can potentially be noticed, altered cell counts can be more difficult to detect, leading to wrong interpretations. Thus, while the scale of the imaging data calls for the use of these semi-automated high-throughput workflows, the complexity of the imaging data necessitates robust quality control (QC) measures to ensure accurate and reliable analysis outcomes that can be reproduced in alignment with the principles of FAIR (findable, accessible, interoperable, reusable) data (Wilkinson et al., 2016).

For researchers, implementing QC allows for identifying required adjustments in experimental protocols at the early stage of the project and increases the trustworthiness of the analysis outcome. Importantly, standardized storage of QC data also enables effective querying of spatial atlases [such as WebAtlas (Li et al., 2023)] and public image repositories (Williams et al., 2017; Hartley et al., 2022). With QC metrics stored in a queryable manner for each dataset, researchers can rapidly access appropriate datasets without the need for visually inspecting raw data and their analysis outcome to decide upon data reuse, thus facilitating benchmarking efforts and allowing for easier training of large-scale models.

However, integrating the generation and storage of QC data into image analysis workflows is challenging, particularly in the absence of tailored software tools and established standard metrics. Therefore, most researchers end up relying on a time-consuming and subjective QC evaluation by manual visual assessment of images using common image viewers such as Fiji, napari, QuPath, Vitessce and Imaris (Schindelin et al., 2012; Bankhead et al., 2017; Keller et al., 2021; Ahlers et al., 2023).

Here, we reflect on the concept of QC in multiplexed image analysis, provide a perspective on its challenges with respect to integration in automated workflows, and discuss potential ways to tackle these challenges.

## 2 What constitutes rigorous QC?

Rather than giving a literal definition, we describe rigorous QC by its implications. Rigorous QC should primarily lead to a reliable analysis outcome, where the underlying limitations of the data are transparent and accessible as part of the final data. Hence, a reliable analysis outcome does not necessitate perfect image quality and flawless processing and analysis of the data, but the interpretation of the analysis results should be done with a very well-argued level of certainty that could be derived from the QC process. For example, what percentage of the cells in the tissue section passed the QC criteria and were included in a particular analysis? Was this number enough to be representative of the tissue?). If metrics such as the number of undersegmented or accurately annotated cells can be estimated for the regions of interest, it would be possible to assess the level of certainty regarding the spatial statistics extracted from the data by making the limitations of the dataset and the analysis clear.

**FIGURE 1:**
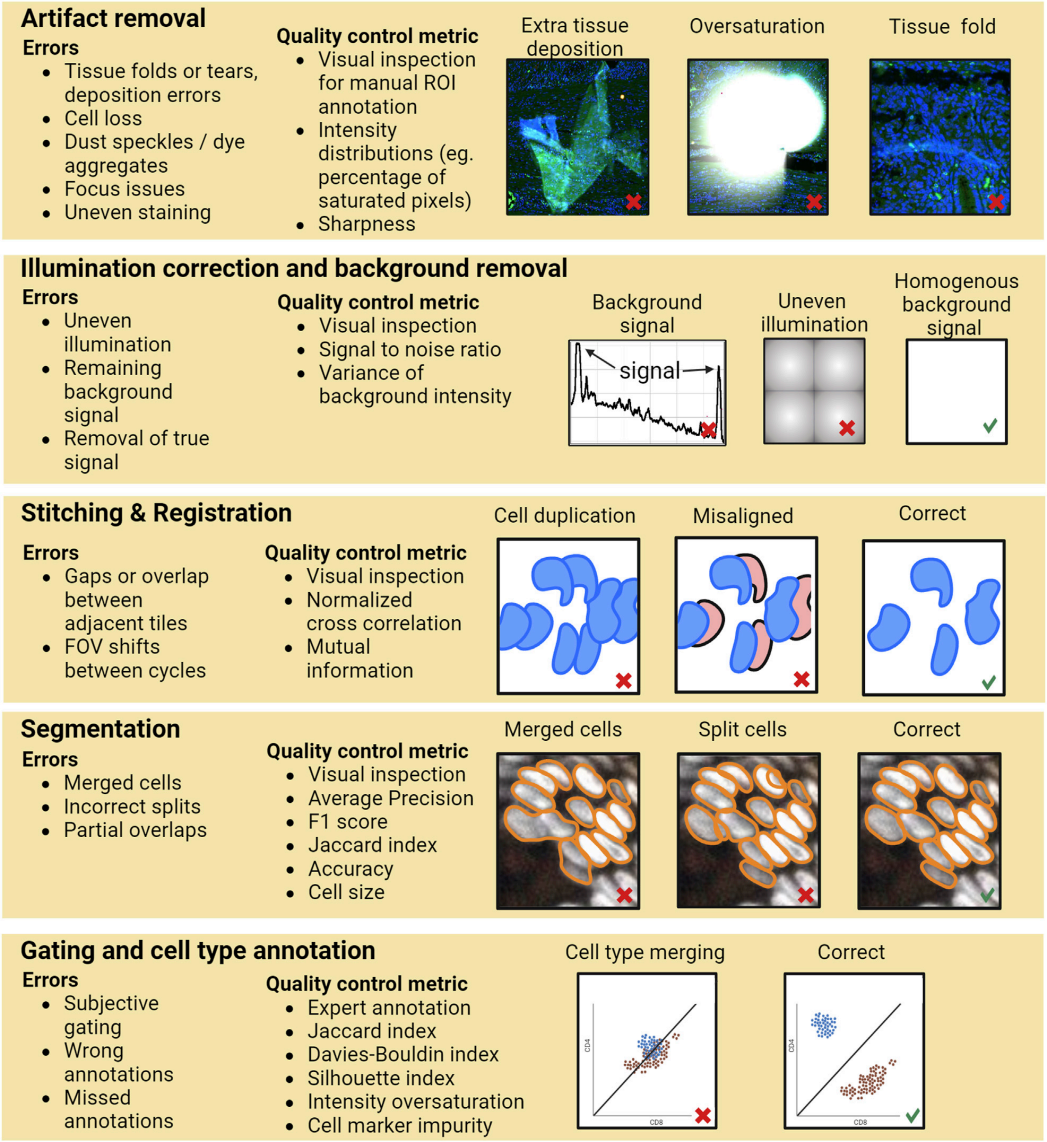
A schematic of an example data processing pipeline for multiplexed protein imaging by immunofluorescence. For each step potential error types and quality control possibilities are exemplified. Calculation of a QC metric would typically require prior annotation of the data. Image data for the artifact removal and segmentation example taken from Human Tumor Atlas Network (Rozenblatt-Rosen et al., 2020).

Currently, most of the researchers perform QC by general visual inspection of the images and/or evaluating the resulting single-cell level data generated by the image analysis pipeline. However, performing QC only based on the final outcome of the whole pipeline may not be sufficient since errors in the various pipeline steps can propagate without necessarily becoming apparent in the final analysis results. For example, if there are tissue artifacts that primarily affect certain anatomical structures heterogeneously present in the sample, such as focus deformations or partial loss of cells from the more fragile lumenal parts of an intestinal section, which would cause segmentation or annotation errors, these might lead to wrong conclusions about the distribution of cell types due to technical biases and errors accumulated during processing. Hence, a rigorous QC involves inspection, evaluation, and storage of traceable information for various steps of the image analysis workflow, including identification and documentation of artifacts such as areas with folded tissue, tissue loss, focusing errors, and other elements that need to be excluded from the analysis (see Figure 1 for an example pipeline with errors and possibilities of QC steps). Having QC at various steps in the pipeline ensures that errors do not go undetected and are not propagated or snowballed into unsupported conclusions. As a simple example, stitching issues in critical areas can lead to hybrid cells or segmentation errors (like a tumor cell and immune cell segmented as one cell), which might lead to errors in cell type annotation, and potentially wrong interpretations of the data. Similarly, insufficient background removal can lead to errors while gating to assign cell type states.

Ideally, the QC readouts would be fully automated, though metrics and tools to enable that are currently absent for many of the processing steps including artifact detection and segmentation. Therefore, it is necessary to store the researcher’s manual assessments in the form of annotations, so that they are taken into account for calculation of the QC metrics. As such, a more transparent QC readout can be established while allowing for a certain degree of subjectivity and manual annotation.

To ensure that the full context of the data is understood and the data is reusable, it is also imperative that the experimental metadata is stored in an easily accessible and queriable manner accompanying the images to ensure that the data can be re-analyzed. For example, the measured intensity levels in the image data are dependent on the way the images were acquired. Making sure that this information is properly stored can be particularly important for investigating batch effects between samples. Hence, we should consider metadata recording also as an integral part of QC, and store the information along with the imaging data and QC data of the processing during both the research and data deposition phase. Ideally, viewing configurations that were used to visualize the outcomes of the various processing steps of the processing and analysis pipeline would also be included to reduce the interobserver variability due to different image viewer settings for manual inspection and visualization.

Taken together, to enhance the transparency, reproducibility and utility of multiplexed imaging or imaging-based spatial omics data, we advocate for increasing the ease and standardization of the QC process which covers manual and automated inspection of the data and documentation of the steps, associated metrics and results throughout various processing steps and image analysis workflow, including the storage of experimental metadata and the QC information along with the data and analysis results in an easily accessible manner.

## 3 Current QC efforts

Perhaps the largest QC effort for imaging data with >500 contributors is Quality Assessment and Reproducibility for Instruments & Images in Light Microscopy (QUAREP-LiMi) (Boehm et al., 2021), which entails multiple working groups dealing with quality assessment of: various features of the microscopy setup; imaging data provenance and QC metadata; image quality, image visualization, and analysis. Notable contributions include guidelines for accurate reporting of common fluorescence light microscopy modalities as well as the Micro-Meta App for extraction and collection of relevant microscope instrument data.

On the practical side, CellProfiler offered one of the early opportunities to incorporate QC in image analysis workflows (Bray et al., 2012) by introducing a threshold on the QC metrics for focal blurring and image saturation to exclude problematic images from analysis. More recently, the AstroPath platform, an end-to-end pathology workflow, incorporated multiplexed immunofluorescence assay development and protocols for the acquisition of the images and a single-marker phenotyping approach aimed at improving the quality of generated data (Berry et al., 2021), by performing a systematic analysis of potential sources of error and providing a pipeline to ensure that optimal experimental settings are applied (e.g., fields of view should have at least 20% overlap when performing a whole slide scan to allow for proper stitching).

With respect to multiplexing-specific metadata, the Minimum Information about Highly Multiplexed Tissue Imaging (MITI) standard has been developed (Schapiro et al., 2022) to serve the need for data and metadata standards for highly multiplexed imaging that are conformant with FAIR standards. The field names in MITI are harmonized with efforts such as QUAREP-LiMi, the Resource Identification Initiative, and antibody standardization effort by the Human Protein Atlas in addition to being compliant with Recommended Metadata for Biological Images initiative (REMBI) (Bandrowski et al., 2016; Edfors et al., 2018; Boehm et al., 2021; Sarkans et al., 2021). Importantly, this standard includes clinical, biospecimen, cell, and cell state metadata topics as well critical information for multiplexed imaging data such as channel metadata (e.g., antibody name, cycle number, fluorophore etc.).

Yet, while these efforts provide a metadata standard and tools to extract and collect the metadata, they are not broadly incorporated in highly multiplexed imaging data generation and analysis workflows, and the QC data is not integrated in the data processing steps. This makes it challenging for the individual researcher to implement the standards when generating and using the data.

Recent efforts aim to address this by directly integrating multiple QC steps into image analysis workflows. For example, CyLinter is an open-source QC software written in Python using the interactive, multidimensional viewer, napari (Baker et al., 2023a; Ahlers et al., 2023) and can also be run as part of the MCMICRO pipeline (Schapiro et al., 2021). It provides a QC workflow via modules to: i) positively or negatively select regions of interest, ii) filter out out-of-focus and counterstain-oversaturated cell nuclei, iii) remove those cells that have shifted or become detached from the slide and iv) define channel intensity cutoffs to exclude cells with abnormal intensities and rescale channel intensities of remaining cells. However, the individual modules require interaction of the user with the whole channel images individually, and as a result, the implementation is not efficient when scaled up for a high number of channels and samples. Also, manual cutoffs such as gating on cell area or intensity are not always stored in a standardized format, making the data stored not easily accessible for non-computational researchers.

A major visual QC effort for imaging-based spatial omics data is TissUUmaps3 (Behanova et al., 2023; Pielawski et al., 2023), which is a browser-based tool for fast GPU-accelerated visualization and exploration of large-scale image data using plugins. It includes image converters for a range of image formats to be converted to a pyramidal format that stores the image at multiple resolutions which can be loaded depending on the zoom level and hence provide a memory-efficient, fast image visualization capability. The StainV&QC plugin enables rapid assessment of intensity normalization by checking proper alignment of the low-dimensional representation of intensity-based features, extracted after segmentation. If the low-dimensional representation of these features align for two samples, the stainings of the two samples are comparable. The ClassV&QC plugin makes it possible to investigate mismatches between cell classification approaches. In the test case, the expert cell classification annotation was compared to the output of a fully convolutional neural network. In case of a mismatch the user can click on cell annotation overlayed on top of an image representing the cell classification of both classification approaches to view patches of the cell in individual channels. The user can subsequently choose which classification is correct. Lastly, the InteractionV&QC plugin allows quick visualization of cellular interactions. With these plugins TissUUmaps3 offers a great visual QC capability. Although they currently do not support extraction of quantitative QC results as the acceptable quality may be defined subjectively and may differ depending on the use case (Behanova et al., 2023), enabling the extraction of such measures could foster transparency about current quality and initiate a discussion on what should be defined as acceptable quality for output of processing.

While these efforts provide valuable standards and tools to lead the way for rigorous QC and data organization, there are still several challenges to integrating these readily into semi-automated image analysis pipelines and making image data truly FAIR.

## 4 Challenges and potential solutions for integrating interpretable QC in image processing pipelines

Challenges for integrating QC in image analysis pipelines and FAIR image data deposition revolve primarily around data format and storage, standardization, automation and scalability.

### 4.1 Image data formats and frameworks

Current QC efforts use a variety of complementary formats. Commonly, the popular TIFF format is used for storing images, with the associated segmentation masks stored as separate unlinked TIFF files and annotations stored in separate csv or tsv files. This does not allow for storing QC data in a manner in which the image and associated analysis data are linked. A unified interoperable format allowing storage of image data and associated analysis data would provide the means of interacting with the data in an integrated manner. This makes it easier for different researchers to interact with the data.

In order to provide a specification for a unified way of storing the data, Moore *et al.* started the development of OME’s next-generation file format (OME-NGFF) (Moore et al., 2021). This is a specification to which the broader bioimage analysis community contributes. It supports a wide range of image data and has a flexible, comprehensive metadata structure. The specification allows for chunking binary pixel image data into smaller parts that are stored in smaller files which supports multi-resolution performance, parallelization, and rapid access when storing image data in the cloud. Furthermore, it allows for associated image analysis data such as segmentation masks to be stored with the image data in a standardized manner. Hence, OME-NGFF offers great potential for integrating QC. Currently, it has an implementation, OME-Zarr, using the Zarr file storage format for chunked, compressed N-dimensional arrays (Miles et al., 2020; Moore et al., 2023). It is based on an open-source specification.

Software frameworks that aim to provide technology agnostic APIs for spatial omics image data are crucial for all the relevant data elements to be kept together in an accessible manner. Many existing frameworks offer great starting points (Palla et al., 2022; Righelli et al., 2022; Couto et al, 2023; Marconato et al., 2023; Moses et al., 2023) but lack certain requirements for comprehensive handling of spatial omics data, such as representation of annotation elements like polygons, support for large images and facilitation of data transformation to a common coordinate framework. For example, emObject is a framework that provides the ability to link annotation tables to segmentation masks (Baker et al., 2023b). However, it does not yet provide the ability to store transformation matrices which would allow transformations to different coordinate spaces to be applied on-the-fly as specified in the OME-NGFF specification. Giotto Suite, a framework embedded in the R ecosystem was also recently released and represents spatial omics data efficiently and in a technology-agnostic manner (Chen et al., 2023). It includes tools to process, analyze, and visualize spatial omics data at different scales and resolutions, though also currently does not build on top of the OME-NGFF community specification.

Another recent effort, SpatialData framework, builds upon the OME-NGFF specification and extends it, and supports representation of image datasets using elements that are common between many image analysis pipelines, namely, images, labels, points, shapes polygons and tables (Marconato et al., 2023). It can also store coordinate transformations along with the data. This, for example, facilitates the alignment of images across imaging rounds without duplicating the image data. Furthermore, it allows spatially querying the data in coordinate systems, offers a plugin for viewing the data in napari and a library for plotting, and provides an interface to the deep learning framework Pytorch. The framework is actively being improved to provide additional processing and analysis capabilities and reduce the required time for analysis.

Frameworks as described above can provide the means for integrating QC in semi-automated workflows by providing spatial biology data abstractions required to represent this type of data.

### 4.2 Standardization and metadata

Another primary challenge for integrating QC into image analysis pipelines is the lack of standardized approaches to perform QC. Typically, researchers examine their data to a certain extent, but their annotations and assessments of quality are usually not stored in a standardized manner.

To enable a more standardized approach to QC, it would be ideal to add and store the relevant annotations in a standardized manner. For this, a QC vocabulary or ontology describing the QC metrics for the processing steps (i.e., which measure is used to determine the accuracy of a certain processing steps and in how it is applied) is required and ideally implemented. The vocabulary should describe the quantitative QC metrics for the processing steps and be implemented in a variety of programming languages to be interoperable. Otherwise, it becomes challenging to transparently report the process of QC and allow the data to be queried. Providing a clear and comprehensive account of how QC has been performed and its outcomes is essential for building trust in the image analysis outcome. It also allows researchers to easily revisit or query their own data, particularly when the number of samples and channels increases.

The same requirements also apply to experimental metadata. Currently, OME-NGFF does not have a specification for storing experimental metadata. A shared challenge is how to author a vocabulary or schema which can be exported in various formats and for which validators can be easily created in multiple programming languages.

A potential solution for this is provided by LinkML, which enables authoring data schemas in a simple format, YAML (Solbrig et al., 2023). The LinkML framework makes it possible to translate these schemas into other frameworks such as JSON-LD and SQL (the former commonly used to store metadata and the latter for relational databases). Furthermore, Python or Java classes can be generated from the schema letting software implementations in these languages to easily ingest and validate the data. By providing an interface to multiple frameworks it potentially facilitates rapid development of implementations for example the OME-NGFF specification in various programming languages.

### 4.3 Semi-automation and scalability

Current QC efforts often rely on extensive visual inspection and manual assessments which may be subjective and time-consuming, especially when dealing with large datasets and numerous image channels as in highly multiplexed imaging.

**FIGURE 2:**
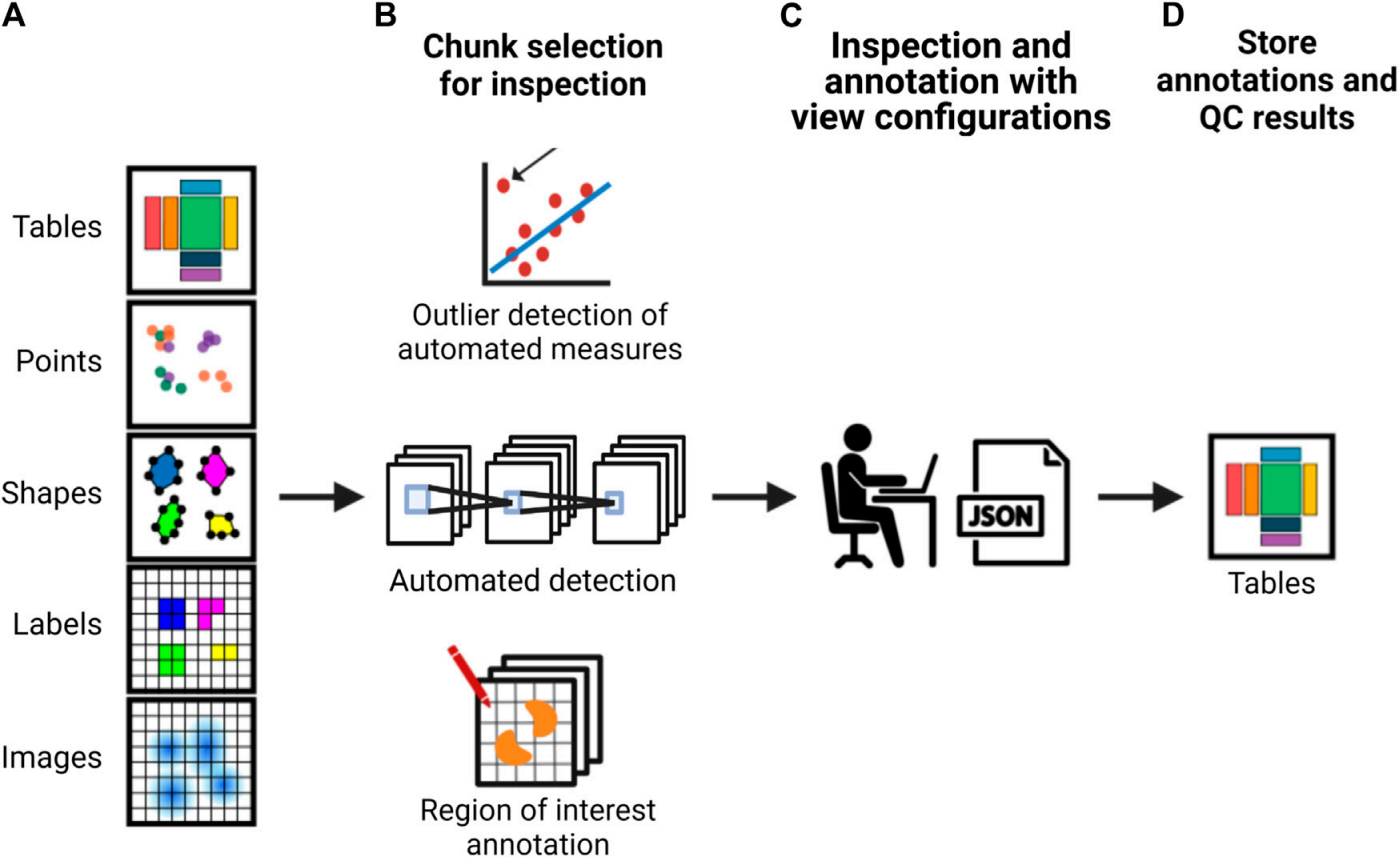
A schematic of an example strategy for implementing more scalable QC in an image analysis pipeline. From left to right, **(A)** a framework provides an interface to the image data and associated analysis data which is used to either **(B)** calculate automated measurements with subsequent outlier detection, use machine learning methods for automated detection or allow the researcher to perform visual inspection or manual annotation using whole images or semi-random or pre-classified crops. This is used to **(C)** automatically create a viewer configuration that can be used to readily view and annotate the data. These view configurations can be altered by the researcher. **(E)** Subsequently, the interface provides the possibility to optionally calculate appropriate QC measures based on annotations and store both the result and the annotations together with the image data and metadata.

The development of the previously described formats and accompanying application programming interfaces (APIs) would lead to more scalable strategies to integrate QC (as exemplified in Figure 2). Researchers would be able to annotate their data using standardized vocabularies and store their annotations alongside the image data. As interacting with large imaging data such as whole slide scans is not efficient, it is expected that a sampling strategy where the researcher would mostly interact with crops of images for visual inspection or determining the quality of the output of a particular processing step would be needed and could be facilitated through an interactive API and viewer. When random crops are used for this process, how many crops are required to estimate the quality of a particular processing step would depend on the heterogeneity of the sample and the data. For example, segmentation quality can differ in dense *versus* sparse cell regions. Hence, ideally, pre-defined measures such as intensity distributions and sharpness could be automatically calculated per image tile or chunk to potentially flag potentially problematic areas for inspection by the researcher, to ensure potential artifacts are inspected and heterogeneity of the sample is well-represented. Other features calculated based on the results of processing steps, such as cell density after segmentation, could also be used to pre-classify the crops to ensure regions with different properties are sampled adequately for visual inspection.

Scalability of QC can be further increased by reducing the annotation burden of the researcher. While certain QC metrics demand extensive user annotation, like those requiring ground truth segmentation, others necessitate less interaction with the data. For example, a qualitative assessment of the segmentation (e.g., assigning classes, for example, incorrectly and correctly segmented) reduces the burden of manual annotation as it may be quicker than performing a detailed ground truth segmentation. The burden could be further reduced when such an annotation strategy is combined with the pre-classified crop sampling strategy described above. By adopting this strategy, summary statistics representing the overall quality of a processing step can be computed; for example, the percentage of correctly segmented cells after segmentation. This requires a calculation of the estimated number of required annotations to represent the data. While in this case the quantitative QC depends on subjective qualitative annotations of the researcher, the approach minimizes the amount of required interaction with the images, allows revisiting annotations by other researchers and may be more informative for the data user.

As the dimensionality of the data increases, composite images become uninformative and visual inspection becomes difficult, necessitating a multichannel view with synchronized cameras in which all individual channels or subgroups of channels are displayed in their own respective view boxes. This allows quick general inspection and annotation of all channels. Although there are many viewers for visualization, QuPath is one of the only open-source viewers to provide this full functionality (Bankhead et al., 2017). Preset viewer configurations (i.e., configurations indicating what channels are grouped in multichannel view and what contrast limits are applied for optimal visual representations) that can be created in a pipeline to allow for readily visualizing specific crops of the data with customized settings would be a very useful addition to such viewers. Combination of automated QC measures, with the option to perform efficient manual annotation/validation by visual inspection using predefined view configurations would be instrumental to make QC efforts more scalable. This also increases the efficiency of viewing the data interactively or a narrative web viewer, such as Minerva story, after analysis has been completed (Hoffer et al., 2020).

## 5 Discussion

Despite many ongoing efforts, the current landscape of QC in multiplexed imaging data analysis presents significant challenges for integration of QC into semi-automated pipelines, primarily due to a lack of standardization of storage formats and QC metrics, and APIs and viewers that seamlessly interface with the data. The absence of a standardized format for data storage also exacerbates the difficulty in revisiting and querying image- and associated data. Advancements in this field could be greatly accelerated by adopting a unified format that abstracts spatial omics data, coupled with APIs and viewers that allow efficient interaction with the data. Furthermore, the development of a standard QC vocabulary is needed to establish APIs that can uniformly execute and record QC processes. On the practical side, to reduce the burden of labor-intensive visual inspection and annotation, there is a pressing need for pipelines capable of generating viewer configurations dynamically. Such configurations would enable image viewers to display and annotate entire images or selected sections in a multichannel view as needed, thereby streamlining the analysis workflow.

Integrating QC in the analysis pipelines is important to minimize the risk of generating inaccurate conclusions from the data. With the increasing availability of automated commercial platforms, researchers often lack access to raw data or the results of individual processing steps. Small, undetected errors in the pipeline can lead to error propagation and misinterpretation of the spatial context (for example, off-target stainings in spatial transcriptomics data - Hartman and Satija, 2024). Integrating QC into image analysis pipelines and standardizing the storage of the resulting data would facilitate a transparent evaluation of data quality, preventing the ingestion of low-quality data by algorithms and machine learning models. Making QC measures readily available alongside image data would also diminish the effort required to use repositories for reliable reference data or to access large datasets of pre-assessed quality for training future machine learning models, potentially reducing the reliance on labor-intensive visual inspection and annotation. This approach would significantly contribute to the reliability and effectiveness of spatial omics analysis, fostering advancements in the field.

We believe that the frameworks mentioned above, with their unique and overlapping capabilities, are instrumental in starting to integrate QC into semi-automated workflows and can represent significant advancements to improve the transparency and utility of the QC process.

We hope that the attention towards including QC measures will not only facilitate the development of automated QC processes, but also lead to longer data lifetimes and improve the usefulness of the data and image data repositories.

## Data Availability

The original contributions presented in the study are included in the article, further inquiries can be directed to the corresponding authors.

## References

[B1] Ahlers J. Althviz Moré D. Amsalem O. Anderson A. Bokota A. Boone P. (2023). *napari: a multi-dimensional image viewer for Python*. Zenodo. 10.5281/ZENODO.3555620

[B2] Alexandrov T. Saez-Rodriguez J. Saka S. K. (2023). Enablers and challenges of spatial omics, a melting pot of technologies. Mol. Syst. Biol. 19 (11). e10571. 10.15252/msb.202110571 PMC10632737 37842805

[B3] Baker E. Mayer A. Trevino A. E. (2023a). emObject: domain specific data abstraction for spatial omics. J. Immunotherapy Cancer (11). 10.1136/jitc-2023-SITC2023.0899

[B4] Baker G. J. Novikov E. Zhao Z. Vallius T. Davis J. A. Lin J. R. (2023b). Quality control for single cell analysis of high-plex tissue profiles using CyLinter. bioRxiv, 565120. 10.1101/2023.11.01.565120 PMC11621021 39478175

[B5] Bandrowski A. Brush M. Grethe J. S. Haendel M. A. Kennedy D. N. Hill S. (2016). The Resource Identification Initiative: a cultural shift in publishing. Neuroinformatics 14 (2), 169–182. 10.1007/s12021-015-9284-3 26589523 PMC5072392

[B6] Bankhead P. Loughrey M. B. Fernández J. A. Dombrowski Y. McArt D. G. Dunne P. D. (2017). QuPath: open source software for digital pathology image analysis. Sci. Rep. 7 (1), 16878. 10.1038/s41598-017-17204-5 PMC5715110 29203879

[B7] Behanova A. Avenel C. Andersson A. Chelebian E. Klemm A. Wik L. (2023). Visualization and quality control tools for large-scale multiplex tissue analysis in TissUUmaps3. Bio. imaging 3 (e6). 10.1017/s2633903x23000053 PMC10936381 38487686

[B8] Berry S. Giraldo N. A. Green B. F. Cottrell T. R. Stein J. E. Engle E. L. (2021). Analysis of multispectral imaging with the AstroPath platform informs efficacy of PD-1 blockade. Science 372 (6547), eaba2609. 10.1126/science.aba2609 PMC8709533 34112666

[B9] Boehm U. Nelson G. Brown C. M. Bagley S. Bajcsy P. Bischof J. (2021). QUAREP-LiMi: a community endeavor to advance quality assessment and reproducibility in light microscopy. Nat. methods 18 (12), 1423–1426. 10.1038/s41592-021-01162-y 34021279 PMC9443067

[B10] Bray M.-A. Fraser A. N. Hasaka T. P. Carpenter A. E. (2012). Workflow and metrics for image quality control in large-scale high-content screens. J. Biomol. Screen. 17 (2), 266–274. 10.1177/1087057111420292 21956170 PMC3593271

[B11] Chen J. G. Chávez-Fuentes J. C. O’Brien M. Xu J. Ruiz E. Wang W. (2023). Giotto Suite: a multi-scale and technology-agnostic spatial multi-omics analysis ecosystem. bioRxiv, 568752. 10.1101/2023.11.26.568752 PMC12510873 41034612

[B12] Consortium H. BMAP Lin S. Posgai A. Atkinson M. Regev A. Rood J. (2019). The human body at cellular resolution: the NIH Human Biomolecular Atlas Program. Nature 574 (7777), 187–192. 10.1038/s41586-019-1629-x 31597973 PMC6800388

[B13] Couto B. Z. Robertson N. Patrick E. Ghazanfar S. (2023). MoleculeExperiment enables consistent infrastructure for molecule-resolved spatial omics data in bioconductor. Bioinformatics (Oxford, England) 39 (9). 10.1093/bioinformatics/btad550 PMC10504467 37698995

[B14] Edfors F. Hober A. Linderbäck K. Maddalo G. Azimi A. Sivertsson Å. (2018). Enhanced validation of antibodies for research applications. Nat. Commun. 9 (1), 4130. 10.1038/s41467-018-06642-y 30297845 PMC6175901

[B15] Eng J. Bucher E. Hu Z. Zheng T. Gibbs S. L. Chin K. (2022). A framework for multiplex imaging optimization and reproducible analysis. Commun. Biol. 5 (1), 438. 10.1038/s42003-022-03368-y 35545666 PMC9095647

[B16] Greenwald N. F. Miller G. Moen E. Kong A. Kagel A. Dougherty T. (2022). Whole-cell segmentation of tissue images with human-level performance using large-scale data annotation and deep learning. Nat. Biotechnol. 40 (4), 555–565. 10.1038/s41587-021-01094-0 34795433 PMC9010346

[B17] Hartley M. Kleywegt G. J. Patwardhan A. Sarkans U. Swedlow J. R. Brazma A. (2022). The BioImage archive - building a home for life-sciences microscopy data. J. Mol. Biol. 434 (11), 167505. 10.1016/j.jmb.2022.167505 35189131

[B18] Hartman A. Satija R. (2024). Comparative analysis of multiplexed *in situ* gene expression profiling technologies. bioRxiv, 575135. 10.1101/2024.01.11.575135

[B19] Hickey J. W. Neumann E. K. Radtke A. J. Camarillo J. M. Beuschel R. T. Albanese A. (2022). Spatial mapping of protein composition and tissue organization: a primer for multiplexed antibody-based imaging. Nat. methods 19 (3), 284–295. 10.1038/s41592-021-01316-y 34811556 PMC9264278

[B20] Hickey J. W. Tan Y. Nolan G. P. Goltsev Y. (2021). Strategies for accurate cell type identification in CODEX multiplexed imaging data. Front. Immunol. 12, 727626. 10.3389/fimmu.2021.727626 PMC8415085 34484237

[B21] Hoffer J. Rashid R. Muhlich J. Chen Y. A. Russell D. Ruokonen J. (2020). Minerva: a light-weight, narrative image browser for multiplexed tissue images. J. Open Source Softw. 5 (54), 2579. 10.21105/joss.02579 33768192 PMC7989801

[B22] Keller M. S. Gold I. McCallum C. Manz T. Kharchenko P. V. Gehlenborg N. (2021). Vitessce: a framework for integrative visualization of multi-modal and spatially-resolved single-cell data. 10.31219/osf.io/y8thv PMC11725496 39333268

[B23] Li T. Horsfall D. Basurto-Lozada D. Roberts K. Prete M. Lawrence J. E. G. (2023). WebAtlas pipeline for integrated single cell and spatial transcriptomic data. bioRxiv. 10.1101/2023.05.19.541329 39160302

[B24] Lin J.-R. Chen Y. A. Campton D. Cooper J. Coy S. Yapp C. (2023). High-plex immunofluorescence imaging and traditional histology of the same tissue section for discovering image-based biomarkers. Nat. Cancer 4 (7), 1036–1052. 10.1038/s43018-023-00576-1 37349501 PMC10368530

[B25] Marconato L. Palla G. Yamauchi K. A. Virshup I. Heidari E. Treis T. (2023). SpatialData: an open and universal data framework for spatial omics. bioRxiv. 10.1101/2023.05.05.539647 PMC11725494 38509327

[B26] Miles A. Kirkham J. Durant M. Bourbeau J. Onalan T. Hamman J. (2020). zarr-developers/zarr-python: v2.4.0 . *Zenodo* . 10.5281/ZENODO.377345

[B27] Moffitt J. R. Lundberg E. Heyn H. (2022). The emerging landscape of spatial profiling technologies. Nat. Rev. Genet. 23 (12), 741–759. 10.1038/s41576-022-00515-3 35859028

[B28] Moore J. Allan C. Besson S. Burel J. M. Diel E. Gault D. (2021). OME-NGFF: a next-generation file format for expanding bioimaging data-access strategies. Nat. methods 18 (12), 1496–1498. 10.1038/s41592-021-01326-w 34845388 PMC8648559

[B29] Moore J. Basurto-Lozada D. Besson S. Bogovic J. Bragantini J. Brown E. M. (2023). OME-Zarr: a cloud-optimized bioimaging file format with international community support. Histochem. Cell Biol. 160, 223–251. 10.1007/s00418-023-02209-1 37428210 PMC10492740

[B30] Moses L. Einarsson P. H. Jackson K. Luebbert L. Booeshaghi A. S. Antonsson S. (2023). Voyager: exploratory single-cell genomics data analysis with geospatial statistics. bioRxiv, 549945. 10.1101/2023.07.20.549945

[B31] Pachitariu M. Stringer C. (2022). Cellpose 2.0: how to train your own model. Nat. methods 19 (12), 1634–1641. 10.1038/s41592-022-01663-4 36344832 PMC9718665

[B32] Palla G. Spitzer H. Klein M. Fischer D. Schaar A. C. Kuemmerle L. B. (2022). Squidpy: a scalable framework for spatial omics analysis. Nat. Methods 19 (2), 171–178. 10.1038/s41592-021-01358-2 35102346 PMC8828470

[B33] Pielawski N. Andersson A. Avenel C. Behanova A. Chelebian E. Klemm A. (2023). TissUUmaps 3: improvements in interactive visualization, exploration, and quality assessment of large-scale spatial omics data. Heliyon 9 (5), e15306. 10.1016/j.heliyon.2023.e15306 PMC10149187 37131430

[B34] Rajewsky N. Almouzni G. Gorski S. A. Aerts S. Amit I. Bertero M. G. (2020). LifeTime and improving European healthcare through cell-based interceptive medicine. Nature 587 (7834), 377–386. 10.1038/s41586-020-2715-9 32894860 PMC7656507

[B35] Righelli D. Weber L. M. Crowell H. L. Pardo B. Collado-Torres L. Ghazanfar S. (2022). SpatialExperiment: infrastructure for spatially-resolved transcriptomics data in R using Bioconductor. Bioinformatics 38 (11), 3128–3131. 10.1093/bioinformatics/btac299 35482478 PMC9154247

[B36] Rozenblatt-Rosen O. Regev A. Oberdoerffer P. Nawy T. Hupalowska A. Rood J. E. (2020). The human tumor Atlas Network: charting tumor transitions across space and time at single-cell resolution. Cell 181 (2), 236–249. 10.1016/j.cell.2020.03.053 32302568 PMC7376497

[B37] Sarkans U. Chiu W. Collinson L. Darrow M. C. Ellenberg J. Grunwald D. (2021). REMBI: Recommended Metadata for Biological Images-enabling reuse of microscopy data in biology. Nat. methods 18 (12), 1418–1422. 10.1038/s41592-021-01166-8 34021280 PMC8606015

[B38] Schapiro D. Sokolov A. Yapp C. Chen Y. A. Muhlich J. L. Hess J. (2021). MCMICRO: a scalable, modular image-processing pipeline for multiplexed tissue imaging. Nat. methods 19 (3), 311–315. 10.1038/s41592-021-01308-y 34824477 PMC8916956

[B39] Schapiro D. Yapp C. Sokolov A. Reynolds S. M. Chen Y. A. Sudar D. (2022). MITI minimum information guidelines for highly multiplexed tissue images. Nat. methods 19 (3), 262–267. 10.1038/s41592-022-01415-4 35277708 PMC9009186

[B40] Schindelin J. Arganda-Carreras I. Frise E. Kaynig V. Longair M. Pietzsch T. (2012). Fiji: an open-source platform for biological-image analysis. Nat. methods 9 (7), 676–682. 10.1038/nmeth.2019 22743772 PMC3855844

[B41] Schürch C. M. Bhate S. S. Barlow G. L. Phillips D. J. Noti L. Zlobec I. (2020). Coordinated cellular neighborhoods orchestrate antitumoral immunity at the colorectal cancer invasive front. Cell 183 (3), 838. 10.1016/j.cell.2020.10.021 33125896 PMC7658307

[B42] Solbrig H. Moxon S. Unni D. Vaidya G. Hegde H. Duncan D. (2023). LinkML. Zenodo. 10.5281/ZENODO.5703670

[B43] Wang J. Browne L. Slapetova I. Shang F. Lee K. Lynch J. (2021). Multiplexed immunofluorescence identifies high stromal CD68+PD-L1+ macrophages as a predictor of improved survival in triple negative breast cancer. Sci. Rep. 11 (1), 21608. 10.1038/s41598-021-01116-6 PMC8566595 34732817

[B44] Wilkinson M. D. Dumontier M. Aalbersberg I. J. Appleton G. Axton M. Baak A. (2016). The FAIR Guiding Principles for scientific data management and stewardship. Sci. data 3, 160018. 10.1038/sdata.2016.18 PMC4792175 26978244

[B45] Williams E. Moore J. Li S. W. Rustici G. Tarkowska A. Chessel A. (2017). Image Data Resource: a bioimage data integration and publication platform. Nat. Methods 14 (8), 775–781. 10.1038/nmeth.4326 28775673 PMC5536224

[B46] Windhager J. Zanotelli V. R. T Schulz D. Meyer L. Daniel M. Bodenmiller B. (2021). An end-to-end workflow for multiplexed image processing and analysis. Nat. Protoc. 18 (11), 3565–3613. 10.1038/s41596-023-00881-0 37816904

